# The impact of the age of first blood meal and Zika virus infection on *Aedes aegypti* egg production and longevity

**DOI:** 10.1371/journal.pone.0200766

**Published:** 2018-07-26

**Authors:** Martha Thieme Petersen, Isabella Dias da Silveira, Aline Tátila-Ferreira, Mariana Rocha David, Thais Chouin-Carneiro, Liesbeth Van den Wouwer, Louis Maes, Rafael Maciel-de-Freitas

**Affiliations:** 1 Laboratório de Transmissores de Hematozoários, Instituto Oswaldo Cruz, Fiocruz, Rio de Janeiro, RJ, Brazil; 2 Laboratório de Imunologia Viral, Instituto Oswaldo Cruz, Fiocruz, Rio de Janeiro, RJ, Brazil; 3 Laboratory for Microbiology, Parasitology and Hygiene (LMPH), University of Antwerp, Belgium; University of California Davis, UNITED STATES

## Abstract

The impact of senescence and pathogen infection on *Aedes aegypti* life-history traits remains poorly understood. This laboratory study focused on the impact of Zika virus (ZIKV) infection and the age of first blood intake on blood meal and clutch sizes, and more importantly on the egg production ratio per μL of blood. Three groups of ZIKV-infected and uninfected *Ae*. *aegypti* females that received their first blood meal at 7 (young feeders), 14 (mature feeders) and 21 days old (old feeders) were monitored daily for survival and received a blood meal free of ZIKV once a week. The number of eggs laid per female were registered 3–4 days after blood feeding. Infection by ZIKV and age of feeding produced a strong negative impact on survival and oviposition success (*e*.*g*. likelihood of laying at least one egg per gonotrophic cycle). Interestingly, clutch size presented a dramatic reduction on uninfected mosquitoes, but raised from 36.5 in clutch1 to 55.1 eggs in clutch 3. Blood meal size remained stable in uninfected females, while a slight increase was observed for the infected counterparts. In uninfected *Ae*. *aegypti*, egg production was strongly affected by the age of feeding with younger females laying three times more eggs than when older. On the other hand, ZIKV-infected mosquitoes had a constant but low egg production. Overall, mosquito senescence and ZIKV infection had an impact on mosquito egg production by causing a sharp decrease in the number of eggs along the clutches for uninfected mosquitoes and a slight increase for infected mosquitoes. Despite some study limitations, our results contribute to a better understanding of the effects of mosquito aging and pathogen infection on the vectorial capacity of *Ae*. *aegypti*.

## Introduction

In the last decades, mosquito-borne arboviruses have emerged in different regions of the globe causing severe outbreaks on human population. Since the 1970’s, dengue virus (DENV) transmission has shown a 30-fold increase in its worldwide incidence with estimates of around 400 million new infections every year [1,2]. During the late 2000’s, chikungunya virus (CHIKV) became pandemic after reaching the Americas with at least two distinct genotypes: the Asian genotype probably arrived through the Caribbean while the East-Central-South African (ECSA) genotype was first detected in central Brazil [3,4]. In 2014, Zika virus (ZIKV) emerged in Pacific islands and later invaded the Americas, leading to a public health emergency due to its association with microcephaly in newborns [5,6]. Between December 2016 and April 2017, an outbreak outside the endemic region of Brazil resulted in the largest epizootic of jungle Yellow Fever virus (YFV) with 209 deaths and a case-fatality superior to 30% [7].

With the exception of the sylvatic cycle of YFV, which is maintained by New World primates and sylvatic mosquitoes, DENV, CHIK and ZIKV have *Aedes* mosquitoes as their primary vectors [8,9]. The dominant role of *Ae*. *aegypti* as primary vector for these arboviruses can partially be explained by their close association with human dwellings. Females are more likely to obtain energy for their metabolism by blood feeding on human hosts rather than on other vertebrates or from sugar feeding. Around 3–4 days later, females preferentially lay their eggs on a variety of man-made breeding sites in the surroundings of human properties [10–12].

The intensity of disease transmission is partially shaped by alterations in vectorial capacity, which is defined as the total number of potentially infectious bites on humans on a single day [13,14]. For example, dengue transmission intensity is governed by local variations of *Ae*. *aegypti* vectorial capacity parameters [15]. An accurate estimate of the components of vectorial capacity in endemic field settings has proven to be extremely difficult due to the complex and multifactorial effects of clime, landscape, mosquito and host densities, and breeding site availability [16].

Although of paramount relevance, the effects of pathogen infection on the biology of mosquitoes have received relatively low attention so far. Some arboviruses are able to invade several tissues including the mosquito’s brain and are likely to modify its physiology and metabolism. Hence, arboviruses are prone to affect vectorial capacity and the pattern of disease transmission [17,18]. A reoccurring observation noted in several studies is the effect of senescence and pathogen infection on fecundity (*i*.*e*. the number of eggs laid per clutch). Older *Culex quinquefasciatus* females laid less eggs over time, especially after 10-days post-eclosion [19]. A similar pattern was also observed for *Cx*. *tarsalis* [20]. Infection with pathogens worsens the fecundity: the number of eggs laid by *Ae*. *aegypti* females decreased more than two-fold within the first five clutches, and dengue-infected individuals presented a sharper reduction on fecundity over time [21,22]. *Culex tarsalis* infected with West Nile Virus presented a harsher reduction in fecundity compared to an uninfected control group [20]. Moreover, a smaller first clutch was observed in *Anopheles stephensi* fed with a blood meal infected with *Plasmodium yoelii nigeriensis* [23].

The present study investigated i) the effects of the age of first feeding and blood meal size on the fecundity of *Ae*. *aegypti* and ii) whether ZIKV infection produced an additional loss on these life history traits.

## Materials and methods

### Mosquitoes

The mosquito population used in this study was the F0 from a field population previously collected in Urca, a high-income area with high infestation at Rio de Janeiro city, Brazil (-22°57'10.29" S -43°09'35.76" W) [24]. A total of 80 ovitraps were distributed on the peridomestic area (houses and buildings) ~50m apart from each other as a way to guarantee larger genetic variability of mosquitoes. Eggs from all ovitraps were hatched in plastic basins containing 3L of water and yeast extract, after which the larvae were separated in basins with 500 larvae and 3L of water each and fed with Tetramin^®^ every day until pupation. Following emergence, adults were kept under insectary conditions (80 ± 5% humidity and 25 ± 3°C) in cylindrical cages with no more than 800 mosquitoes per cage and fed *ad libitum* with 10% sucrose solution for up to 36h before the first blood meal. Adults were allowed to mate until the females were offered their first blood meal.

### Virus strain

The infections were performed using one of the circulating strains of ZIKV (BRPE243/2015) obtained from a patient’s blood in Pernambuco during the 2015 Brazilian outbreak and since then maintained in cell culture [25]. Viral titers were quantified via plaque-forming assay prior to experimental infection. The virus stock contained 3.55 x 10^6^ PFU/mL and was stored at -80°C until use.

### Experimental design and oral infection with ZIKV

**Fig 1:**
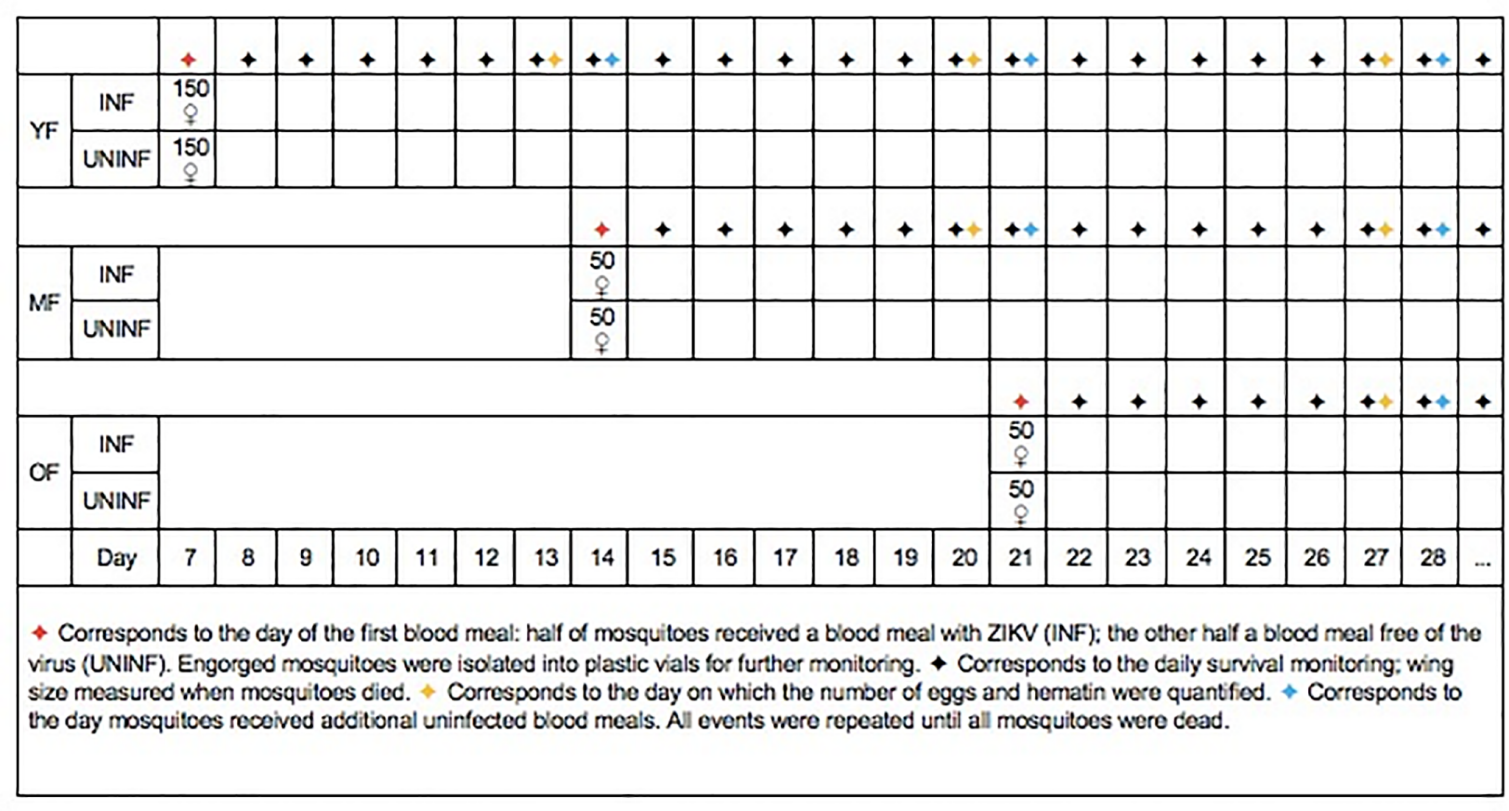
Experimental design: Sample size of each group and monitoring scheme.

To better understand the effects of the age of first feeding on fecundity, adult *Ae*. *aegypti* females were separated into three different groups. Each group received the first blood-meal (infected or uninfected) on either 6–7 (young feeders, YF), 13–14 (mature feeders, MF) or 20–21 days-old (old feeders, OF). At 36h before the first blood meal, mosquitoes were deprived from the sucrose solution and were at this moment divided into two sub-groups: one receiving ZIKV-infected blood in a proportion of 1mL of virus to 2mL of washed human erythrocytes (infected); the other receiving blood mixed with 1mL of cell culture (uninfected). The oral infection was conducted through a membrane feeding system (Hemotek, Great Harwood, UK) adapted with a pig-gut covering. After feeding for 30 minutes, fully engorged females were placed in individual plastic vials containing a piece of humid cotton covered with filter paper as oviposition substrate and covered with mosquito net on the top. A cotton soaked in 10% sucrose solution was provided as carbohydrate source. The same procedure was repeated with MF and OF (Fig 1). Sample size for YF was 300 females (150 ZIKV-infected and 150 uninfected) and MF and OF groups had 100 females each (50 ZIKV-infected and 50 uninfected). A sample of 18 infected mosquitoes was kept in small cylindrical cages until 14 and 21 days post-infection (dpi) and then stored at -80°C to confirm ZIKV infection. When a dead mosquito was observed, it was removed from the plastic vials; wing lengths were measured as the distance from the axillary incision to the apical margin, excluding the fringe [26].

### Blood feeding and fecundity

After the first blood meal, both infected and uninfected females received an uninfected blood meal for 30 minutes once a week. On 4–5 days after every blood meal, the filter papers were removed from the vials and the number of eggs laid per *Ae*. *aegypti* female was recorded. A new filter paper was added as oviposition substrate for the following clutch. These procedures were repeated every week until the mosquitoes from all age groups had died.

### Blood meal size quantification

After recording the number of eggs per female, the filter papers were added to 1.5mL tubes containing 1mL of a 1% lithium carbonate solution as a way to dilute the feces. A standard curve was prepared by diluting known amounts of blood and measuring the corresponding absorbance (0; 0.8; 1.6; 2.4; and 3.2 μL) producing a R^2^ = 0.97. The samples were analyzed in a spectrophotometer with an absorbance at 387 nM [27]. The ratio of eggs produced per blood meal was calculated on the first three gonotrophic cycles by dividing the number of eggs per female that laid at least one egg by the hematin estimation.

### ZIKV-infection confirmation

A total of 18 individuals (10 collected at 14 dpi and 8 at 21 dpi) were used to confirm ZIKV-infection. Viral RNA was extracted from the mosquito whole body using the QlAamp Viral RNA Mini kit (Qiagen, Hilden, Germany) following the manufacturer’s instructions. Detection and quantification of viral RNA was performed using qRT-PCR with SuperScriptTM III PlatinumTM One-Step qRT-PCR Kit (Thermo Fisher Scientific, Invitrogen) in QuantStudio 6 Flex Real-Time PCR System (Applied Biosystems). Each reaction was made using 600 nM forward primer (5’-CTTGGAGTGCTTGTGATT-3’, genome position 3451–3468), 600 nM reverse primer (5’-CTCCTCCAGTGTTCATTT-3’, genome position 3637–3620) and 800 nM probe (5’FAM- AGAAGAGAATGACCACAAAGATCA-3’TAMRA, genome position 3494–3517). The cycling conditions 95°C for 2 minutes, 40 amplification cycles at 95°C for 15s, 58°C for 5s and 60°C for 30s. Virus copy number in each sample was calculated by interpolation from a standard curve made up of a 7-point dilution series of an *in vitro* transcribed ZIKV RNA [28].

### Statistical analysis

*Ae*. *aegypti* longevity presented a non-normal distribution, but the logarithm of longevity satisfied the assumption of normality (Shapiro-Wilk W = 0.9915, P = 0.0592). Day zero was set as the day in which the YF received their first blood meal. Daily survival monitoring for MF and OF started on the day mosquitoes fed. The effects of treatment (infected or uninfected), age on the day of infection (YF, MF, OF) and wing length on the log_10_ of mosquito longevity were analyzed with ANOVA. A log-rank test compared the survival distribution of *Ae*. *aegypti* females from different treatment and age of first feeding. Survival rate is defined as the number of individuals still alive as a function of time.

Fecundity and blood meal size were analyzed by considering the first three clutches of eggs, as only a small number of females (especially OF) blood fed and laid eggs at later clutches precluding adequate numbers for analysis. Two aspects of fecundity were analyzed: oviposition success and clutch size. The oviposition success, *i*.*e*. the likelihood that a mosquito laid at least one egg (at a given clutch) was analyzed with a logistic analysis that included treatment, age of first feeding, wing length and clutch-number (*i*.*e*. age). Next, the number of eggs per clutch was analyzed from those mosquitoes that laid at least one egg, using a repeated measures analysis and square-root transformed the number of eggs to satisfy the assumptions of normality. We included clutch-number as the variable repeatedly measured and estimated the effects of treatment, age of first feeding, wing length and ageing on clutch size. Blood meal size in the first three blood meals was analyzed by repeated measure analysis. Blood meal was included as the variable repeatedly measured and we estimated the effects of treatment, age of first feeding, wing length and ageing on the amount of blood ingested over time. All analyses were carried out with the statistical software JMP 9 (http://www.jmp.com/).

### Ethical statement

Human blood was obtained from anonymous donors whose blood bags would be discarded due to small volume. Blood was derived from the blood bank of the Rio de Janeiro State University. We have no information on donors, including sex, age and clinical condition. The use of human blood was approved by the Fiocruz Ethical Committee (process CAAE 53419815.9.0000.5248).

## Results

### ZIKV oral infection

**Fig 2:**
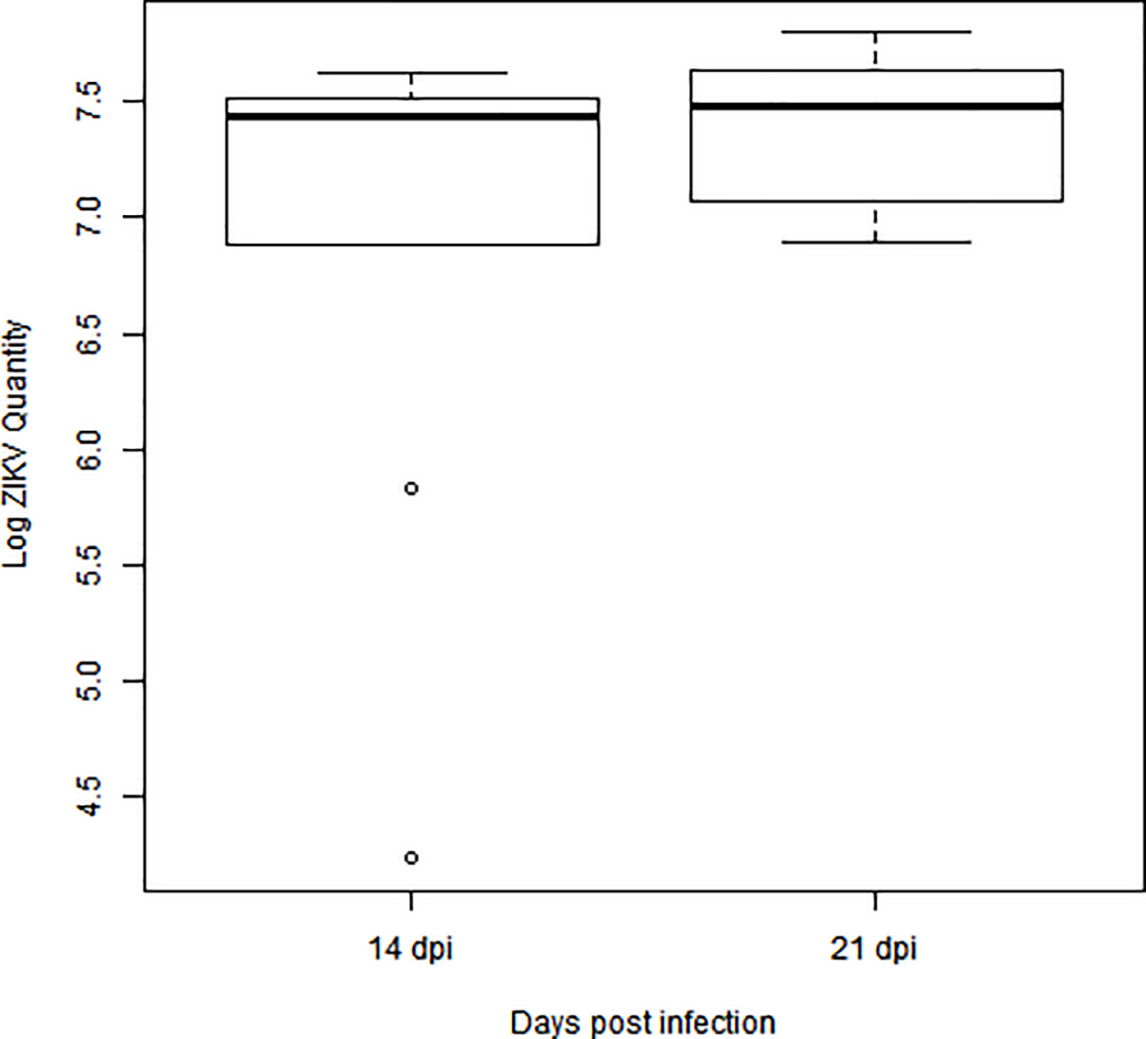
Viral load in the *Ae*. *aegypti* mosquitoes infected with ZIKV. A total of 18 mosquitoes was individually tested for the presence of ZIKV RNA copies with RT-PCR on days 14 (N = 10) and 21 (N = 8) post infection (dpi).

A total of 500 *Ae*. *aegypti* field-caught females from Urca, Rio de Janeiro, Brazil were divided into three groups according to the age when they received their first blood meal: YF (first blood meal at 6–7 days old, N = 300), MF (first blood meal at 13–14 days old, N = 100) and OF (first blood meal at 20–21 days old, N = 100). In each group, half of the mosquitoes received a ZIKV-infected first blood meal, while the other half served as uninfected control receiving only blood and cell culture media free of ZIKV following the same feeding procedure. A sample of 18 mosquitoes was individually tested at 14 (N = 10) and 21 days (N = 8) post infection (dpi) for the presence of ZIKV RNA copies with RT-PCR. All mosquitoes showed high numbers of ZIKV RNA copies (average 2.6 x 10^7^ PFU), confirming infection. Mosquitoes sampled at 14 and 21 dpi had a comparable amount of ZIKV (t-test = -1.28; df = 11.003; p = 0.228; Fig 2) supporting the assumption that the mosquitoes used in the fecundity experiments were also ZIKV positive.

### ZIKV effects on survival

**Fig 3:**
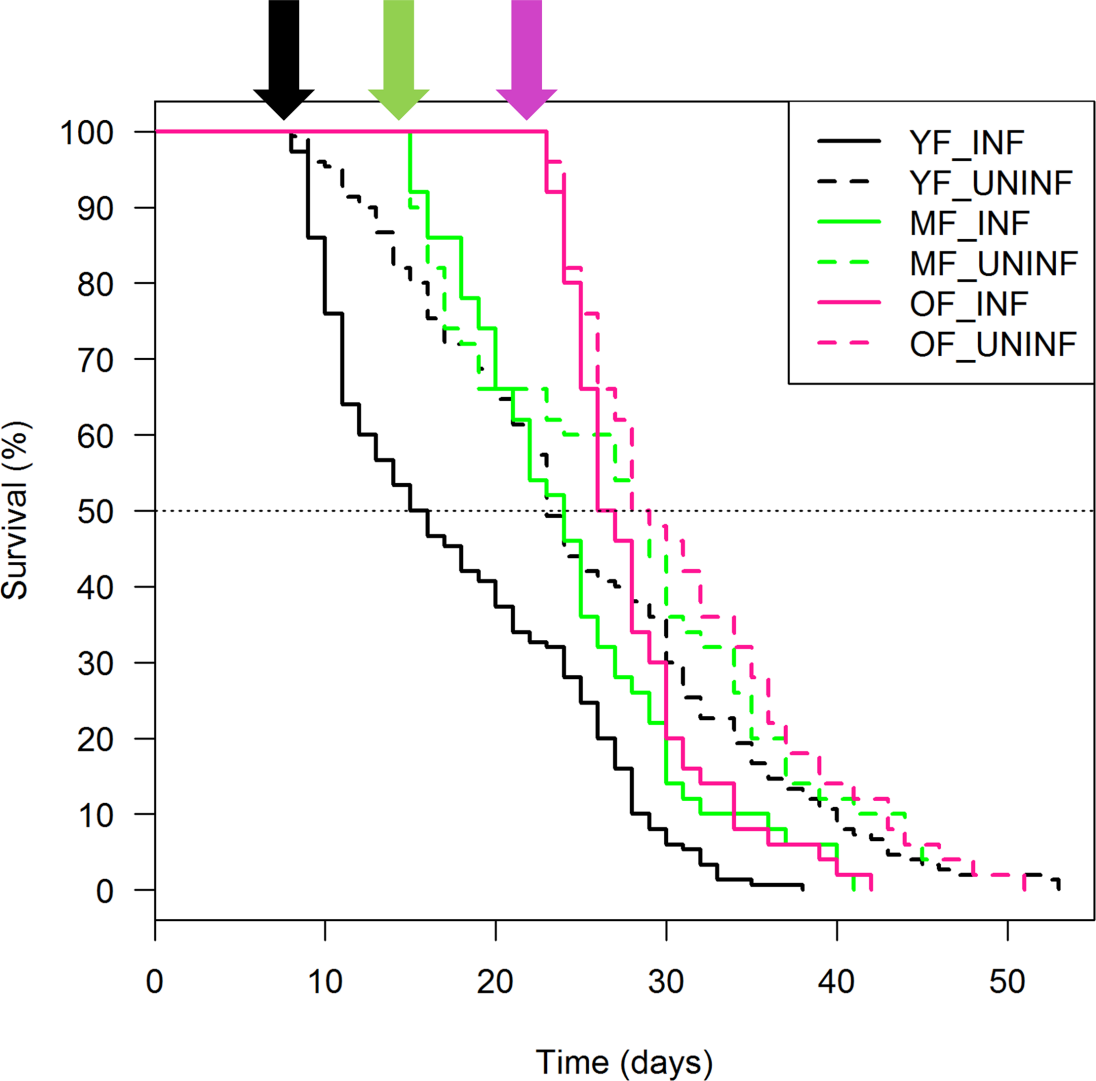
Survival curves of three cohorts of *Ae*. *aegypti* females infected with ZIKV and uninfected counterparts. Data based on the daily monitoring of survival of 500 *Ae*. *aegypti* females: 300 YF, 100 MF and 100 OF. Half of mosquitoes per group was ZIKV-infected. Arrows indicate the day on which each group had it first blood meal.

Regardless the age group, uninfected mosquitoes survived longer than the ZIKV-infected counterparts (YF: χ^2^ = 46.7, df = 1, P < 0.001; MF: χ^2^ = 6.3, df = 1, P = 0.014; OF: χ^2^ = 8.5, df = 1, P = 0.003). Survival curves indicate a sharp decrease in survival immediately after blood feeding, irrespective of the age group and treatment (Fig 3). As expected, survival was also affected by the age of first feeding, since mortality was higher when older mosquitoes were blood fed (Table 1). The ANOVA corroborated the survival data: ZIKV-infected mosquitoes survived less than the uninfected and the age of infection negatively affected survival (Table 2). A strong interaction between treatment and age group was observed: the negative effects on mosquito survival were more evident when older mosquitoes were infected with ZIKV.

**Table 1:** Log-rank p-values of the paired comparison of survival curves of infected and uninfected *Ae*. *aegypti* females from YF (fed with 6–7 days old), MF (13–14 days old) and OF (20–21 days old) groups.

	Uninf_YF	Uninf_MF	Uninf_OF	Inf_YF	Inf_MF	Inf_OF
Uninf_YF						
Uninf_MF	**0.012**					
Uninf_OF	**<0.001**	**0.035**				
Inf_YF	**<0.001**	**0.017**	0.081			
Inf_MF	**<0.001**	**0.014**	0.062	0.575		
Inf_OF	**<0.001**	**<0.001**	**0.003**	**<0.001**	**0.002**	

**Table 2:** Analysis of variance (ANOVA) of the logarithm of survival of ZIKV-infected and uninfected *Ae*. *aegypti* mosquitoes.

Source	d.f.	Sum of squares	*F*	*P-value*
Treatment	1	4.883	36.76	**<0.0001**
Age group	2	1.931	7.27	**0.0008**
Wing size	1	1.315	9.90	**0.0018**
Treatment + age group	2	1.081	4.07	**0.0177**
Treatment + wing size	1	0.069	0.52	0.4701
Age group + wing size	2	0.972	3.66	**0.0265**

d.f.: degree of freedom.

### Oviposition success

Oviposition success in the non-infected group was not affected by the age at which the mosquitoes received their first blood meal. On the other hand in the infected group, the likelihood of females laying at least one egg per gonotrophic cycle was strongly influenced by the age at infection, dropping from a success of 76.1% in YF to 59.3% in OF (Table 3). Regardless of the age group, ZIKV-infected mosquitoes were significantly less likely to lay eggs than the uninfected group.

**Table 3:** Logistic regression analysis of the clutch, treatment, wing size and cohort on the success of oviposition of *Ae*. *aegypti* females.

Source	Nparm	d.f.	χ^2^	P-value
Clutch	6	6	8.702	0.1910
Age group	4	4	15.079	**0.0045**
Wing	2	2	2.752	0.2525
Treatment	2	2	54.868	**< .0001**
Wing + treatment	2	2	0.683	0.7104
Age group + treatment	4	4	0.382	0.9838

Nparm: Number of parameters associated with the effect; d.f.: degree of freedom; χ^2^: chi square test value.

### Fecundity

**Fig 4:**
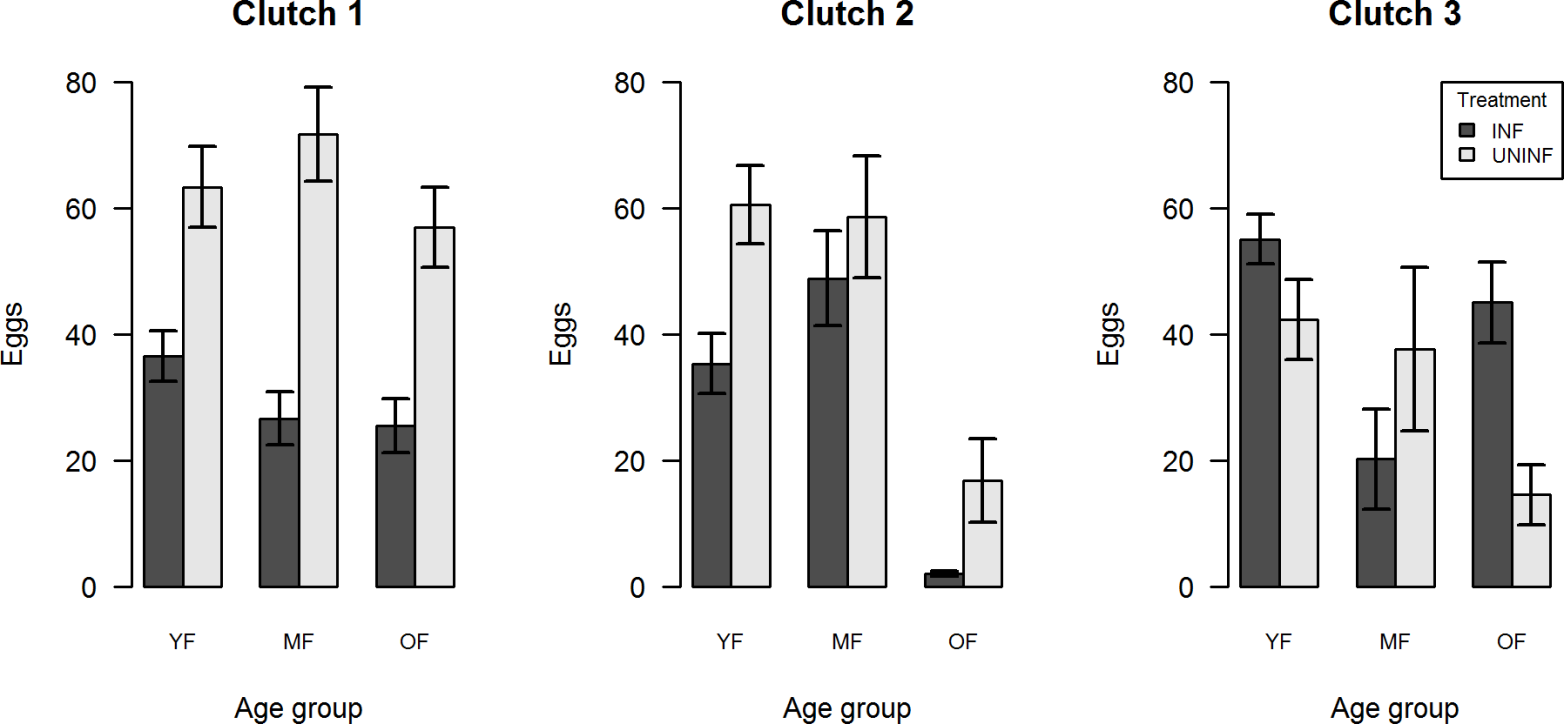
Average number of eggs laid by mosquitoes from the different treatment and age groups. Data based on the weekly observation of ZIKV-infected and uninfected mosquitoes.

This analysis consisted on the number of eggs laid by the females who laid at least one egg. Overall, ZIKV-infected mosquitoes laid less eggs than the uninfected with the exception of uninfected YF which dropped from 63.3 to 42.3 eggs from clutch 1 to clutch 3, while clutch sizes of the infected raised from 36.5 eggs in clutch 1 to 55.1 eggs in clutch 3 (Fig 4). The age of the first feeding apparently had no relevant influence on the number of eggs laid per gonotrophic cycle. Wing size had no significant effect on clutch sizes (Table 4).

**Table 4:** Repeated measures analysis (with clutch size as the repeatedly measured variable) of the square-root of the number of eggs laid by *Ae*. *aegypti* females.

Source	Num df	Den df	*F*	*P-value*
Clutch + treatment	2	53	3.374	**0.041**
Clutch + age group	4	106	0.634	0.638
Clutch + wing	2	53	0.287	0.751

Num df: Numerator degree of freedom; Den df: Denominator degree of freedom.

### Blood meal size

**Fig 5:**
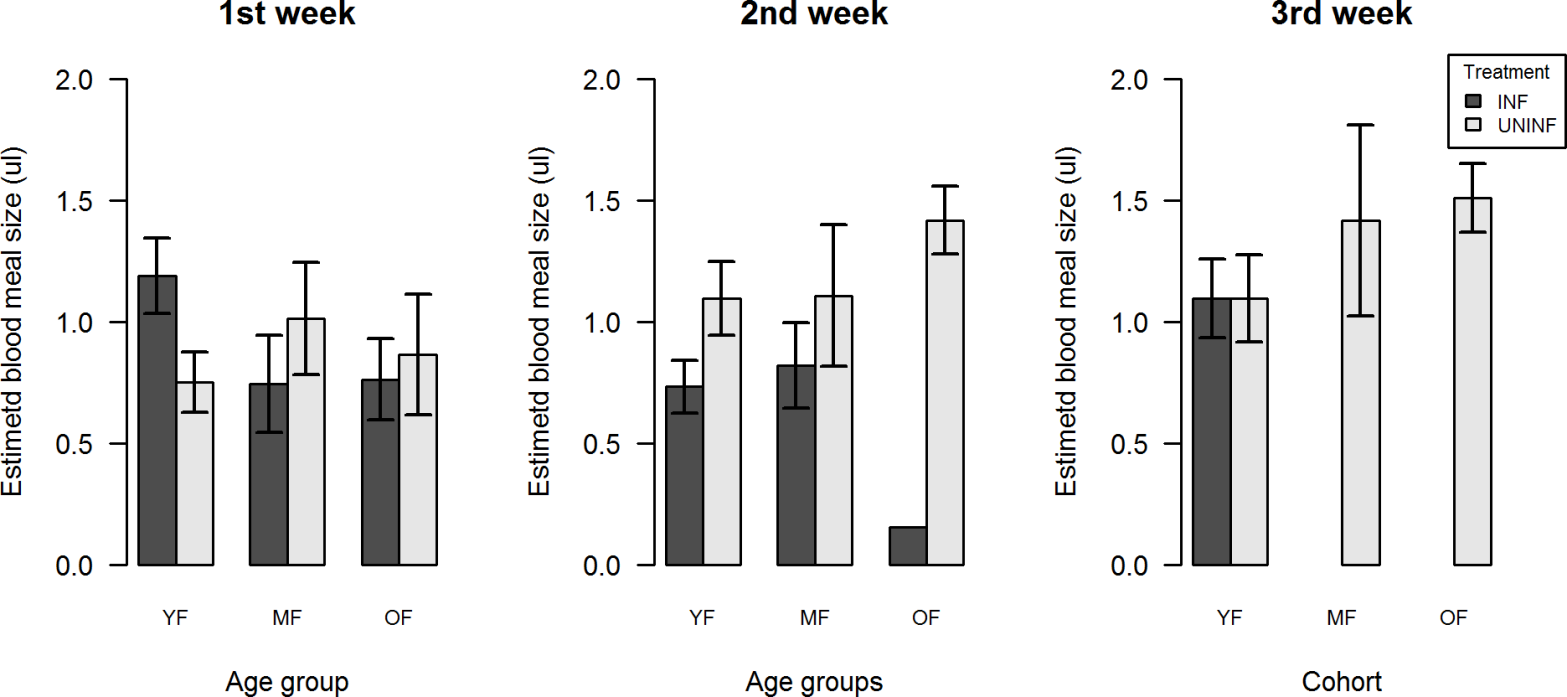
Average blood meal size of the different treatment and age groups. Data based on the weekly quantification of blood meal size of ZIKV-infected and uninfected *Ae*. *aegypti* females.

**Fig 6:**
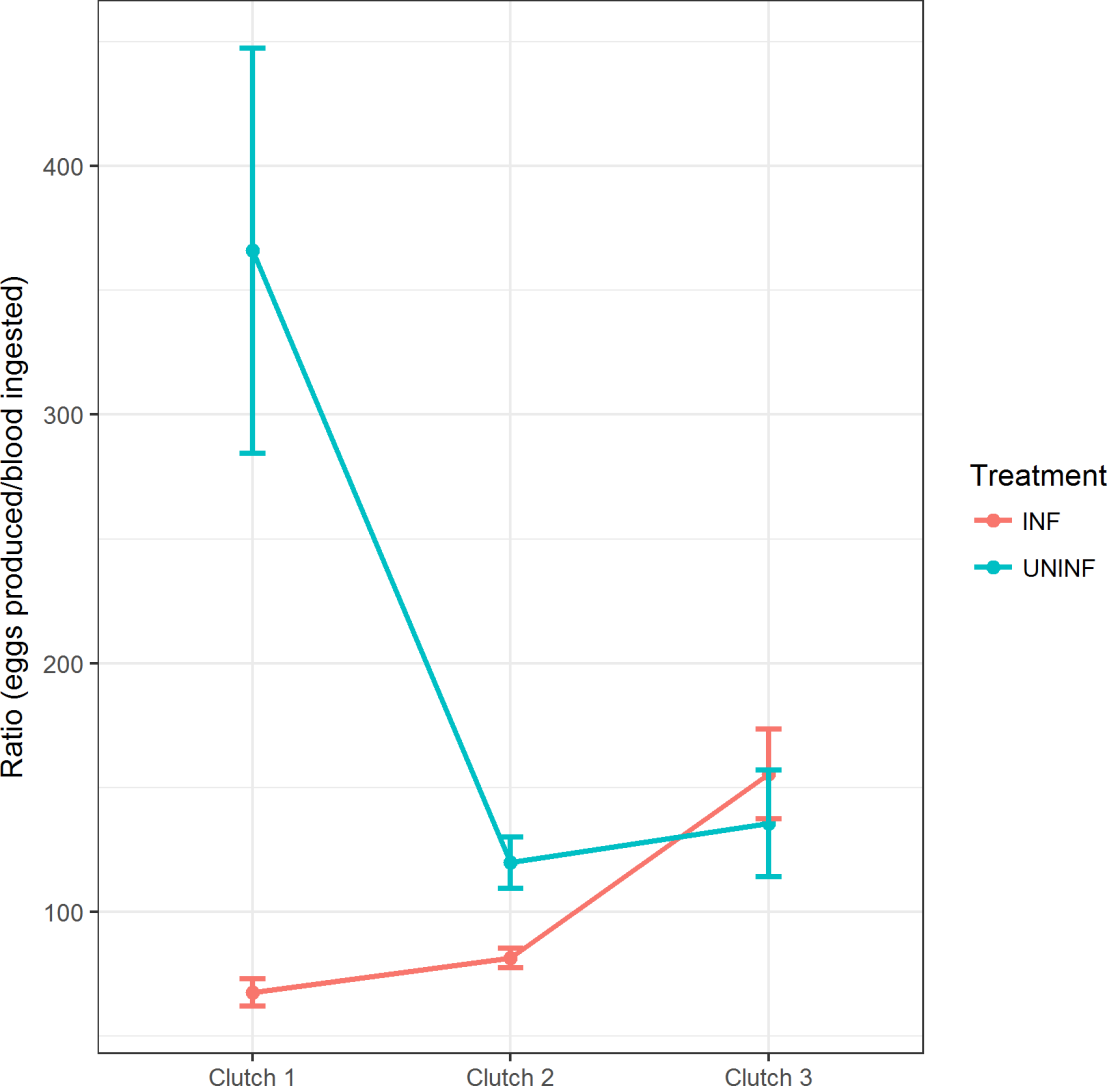
Ratio of egg production per μL of blood of infected and uninfected *Ae*. *aegypti* females. Calculated as the number of eggs laid per female that laid at least one egg divided by the hematin relating to these eggs.

Blood meal size was measured weekly by quantifying the hematin from mosquito feces on the filter paper in the vials (Table 5). ZIKV-infected females ingested significantly more blood than their uninfected counterparts in the first week (*F* = 9.386, df = 1, P = 0.002) (Fig 5). No significant effects were noted for age of infection and wing size. The blood meal size of uninfected mosquitoes remained stable over the two first gonotrophic cycles, however, the amount of eggs produced upon a roughly similar amount of ingested blood dropped with age (Fig 4; Fig 5). The ratio of egg production per blood volume ingested remained constant for ZIKV-infected mosquitoes, while a reduction of egg production over time was noted in the uninfected group. Infected individuals were less effective in producing eggs from a blood meal than uninfected ones in the first two gonotrophic cycles (Fig 6).

**Table 5:** Repeated measures analysis (with hematin as the repeatedly measured variable) of the square-root of the blood meal size taken by *Ae*. *aegypti* females.

Source	Num df	Den df	*F*	*P-value*
Clutch + Treatment	2	65	4.016	**0.022**
Clutch + Age group	4	130	1.203	0.312
Clutch + Wing	2	65	1.421	0.248

Num df: Numerator degree of freedom; Den df: Denominator degree of freedom.

## Discussion

This study investigated in detail the potential impact of aging, blood meal size and ZIKV infection on *Ae*. *aegypti* life-history traits covering fecundity, namely oviposition success, clutch size and egg production per unit of blood ingested. Mosquitoes received their first blood meal (ZIKV-infected or uninfected) at the age of 7, 14 or 21 days-old. ZIKV infection produced a negative effect on lifespan and oviposition success, but increased the number of eggs laid per female at later clutches. Furthermore, egg production presented a sharp decrease over time in uninfected mosquitoes, while ZIKV-infected individuals presented a low but stable production of eggs per μL of blood ingested.

Mosquito survival is one of many parameters that can influence vectorial capacity since mosquitoes must live for at least 10 days after infection to support ZIKV transmission [29]. Our data show that ZIKV consistently caused a negative effect on survival: all three age groups that had received a first infective blood meal presented lower survival rates than their uninfected counterparts. The differences in survival rates between the age groups, infected or not, showed a strong age-dependent factor on mortality [30] that was further enhanced by the presence of ZIKV infection. Mortality was strongly associated with the first blood meal with ~30% of mosquitoes dying in the days following blood ingestion. Interestingly, MF and OF showed similar survival trends after blood-feeding, but ZIKV-infected individuals started to die faster one week after infection (Fig 2). We hypothesize that the cost of eliciting an immune response to ZIKV increases with age, enhancing mortality of OF compared to YF and MF (Table 1). The likelihood of younger mosquitoes presenting a longer lifespan after ZIKV-infection reinforces that arbovirus transmission models must consider a different mortality distribution for infected individuals [30]. More details regarding the age-dependent mortality, particularly in the scenario where disease vectors are infected with their natural pathogens, would incorporate a more comprehensive knowledge on disease transmission [30–33].

ZIKV infection had a significant impact on fecundity. The likelihood of infected individuals laying at least one egg was statistically lower than for their uninfected counterparts. On the other hand, YF infected females laid a bigger 3^rd^ clutch compared to those uninfected. As far as we are aware, there are yet no papers pointing to any modification in mosquito fecundity due to ZIKV infection. DENV infection is able to reduce fertility and fecundity in vertically infected batches [34] as well as the oviposition success and clutch size in orally challenged individuals [21,22]. The age of the first blood meal negatively affected oviposition success but presented no significant effect on clutch size. The number of eggs laid often decreases over time but seems to reduce faster if mosquitoes are infected with pathogens. In *Cx*. *quinquefasciatus*, the number of eggs per clutch reduced significantly as the mosquitoes senesce [19]. A sharper reduction on clutch sizes was detected when *Cx*. *tarsalis* and *An*. *stephensi* were infected with WNV and *P*. *yoelli nigeriensis*, [20,23]. *Ae*. *aegypti* females infected with a DENV-2 strain had lowered fecundity with the main impact occurring 2–3 weeks post-infection [22]. These findings of age-dependent effects on life-history was thought to be a consequence of the dynamics and tropism of DENV, since it is disseminated over the *Ae*. *aegypti* body after ~10–14 days [35]. The biological relevance of the reduction of oviposition success and late increase on clutch size in ZIKV-infected mosquitoes is still unknown.

So far, the fitness cost due to ZIKV infection on *Ae*. *aegypti* mosquitoes remains largely unknown. A cost of arbovirus infection on vector survival was demonstrated in DENV-2 infected *Ae*. *aegypti*, as infected groups showed higher mortality rates than uninfected [21,22]. One important consideration regarding the fitness cost of arbovirus is the natural history of both virus and vectors. Vector competence to a same virus strain often presents great variation among mosquito populations, showing a strong geographical component [36–39]. Here, we used *Ae*. *aegypti* mosquitoes from Rio de Janeiro city and a ZIKV from Pernambuco, a Northeast State distant ~1,800Km. Despite the linear distance between Rio de Janeiro and Pernambuco, ZIKV emerged from an unimportant virus with mild symptoms to a public health emergence in less than a decade, which could mean that the interactions between *Ae*. *aegypti* and ZIKV are too recent for evolution leading to genotype by genotype interactions. Therefore, the impact observed here on mosquito longevity and fecundity is potentially experienced by natural *Ae*. *aegypti* populations with the arrival of ZIKV.

*Ae*. *aegypti* is highly adapted to densely urbanized areas, feeding mostly on human hosts and laying eggs 3–4 days later on man-made breeding sites [10,12,40]. The number of eggs laid per gonotrophic cycle is dependent on the amount of blood ingested [19]. Our results show that uninfected mosquitoes ingested a stable amount of blood on the first three gonotrophic cycles, but the number of eggs produced decreased from 63,3 to 42,3 from clutch 1 to 3 (Fig 3). These data suggest that older mosquitoes become less effective in producing eggs. On the other hand, the blood meal sizes varied in a similar trend over the first three gonotrophic cycles, but there was an increase in the number of eggs for the ZIKV-infected mosquitoes. As a consequence, the ratio of eggs produced per μL of blood ingested exhibit a slight increase for ZIKV-infected and a sharp decrease for uninfected mosquitoes (Fig 5). Although there are still no other studies with observations on feeding behavior for ZIKV-infected individuals, studies on other vector-parasite systems have reported changes on feeding behavior. For example, *Ae*. *aegypti* and *An*. *gambiae* showed an increased bite rate and probing time when infected with *P*. *gallinaceum* and *P*. *falciparum*, respectively [41,42]. Similar results were seen for *Ae*. *aegypti* mosquitoes infected with DENV with increased probing time, larger blood intake [17,22] as well as a higher avidity to start a second blood meal [43]. Studies with *Cx*. *tarsalis* infected with WNV also showed that the infected group would ingest a larger amount of blood than the uninfected group [20].

Although the ZIKV-infected group showed a constant egg production during their lifespan, it was on a lower ratio than the uninfected group (most pronounced in clutches 1 and 2). Not much is known about the effects of immune response in ZIKV-infected *Ae*. *aegypti* models. Our data suggest discrepant effects of infection since it negatively affected mosquito survival rates and oviposition success but surprisingly increased clutch sizes over time. Perhaps, the lower egg production per μL of blood ingested in ZIKV-infected versus uninfected mosquitoes is a manifestation of the fitness cost associated to infection. The presence of ZIKV may likely stimulate mosquitoes to mount an immune response to clear infection, although in-depth knowledge of cellular and humoral immunity responses of *Ae*. *aegypti* to arboviruses is still growing. The presence of midgut infection barriers seems to be the most efficient way to avoid virus dissemination [44]. For instance, RNA interference may modulate infection by producing molecules that inhibit virus replication [45]. Eliciting an immune response may have caused a trade-off with clutch size resulting in a lower egg production per μL of blood ingested [46]. Although our results are relevant, our study design did not address such questions.

Although our results point to a negative impact of Zika virus, the biological relevance of these results may be limited for two reasons. Firstly, *Ae*. *aegypti* PDS (Probability of Daily Survival) ranges around 0.83–0.87 in low income crowded areas and 0.60–0.70 in higher income localities [24,47]. Considering a PDS equals 0.75, only 5.6% of mosquito females would survive longer than the extrinsic incubation period of 10 days to ZIKV [29,48]. As such, only a few mosquitoes would survive long enough to overcome the negative effects of ageing and infection on a field scenario, which is much shorter than observed in lab settings [49]. Secondly, very few mosquitoes in the field are found naturally infected with ZIKV [9], making it unlikely that the fitness cost caused by the virus would have any effect on the natural population.

Our exploration of the effects of ageing and ZIKV infection on the fitness of *Ae*. *aegypti* revealed a strong age-dependent effect in the survival of both groups, in the clutch size of the uninfected mosquitoes and in the oviposition success of the infected group. Additionally, ZIKV had a negative impact on oviposition success and clutch size in the first two gonotrophic cycles. We also showed that ZIKV infected mosquitoes seem to ingest a larger amount of blood during their first meal, which may increase the potential to transmit the virus [50]. The fitness cost associated with ZIKV infection is likely to have an important impact on ZIKV transmission. Further investigations are still required to estimate the impact of arboviruses on mosquito biology in more realistic settings, for example by varying the temperature in which mosquitoes are maintained, varying the virus titer of the initial inoculum and using mosquitoes and virus from the same geographical area. Anyhow, this study is the first to demonstrate the negative impact of ZIKV infection on *Ae*. *aegypti* biology.
